# Complete Genome Sequences of Two Feline Leukemia Virus Subgroup B Isolates with Novel Recombination Sites

**DOI:** 10.1128/genomeA.00036-12

**Published:** 2013-01-15

**Authors:** H. Stewart, O. Jarrett, M. J. Hosie, B. J. Willett

**Affiliations:** Medical Research Council, University of Glasgow Centre for Virus Research, Institute of Infection, Immunity and Inflammation, College of Medical, Veterinary and Life Sciences, University of Glasgow, Glasgow, United Kingdom

## Abstract

It is generally accepted that all primary isolates of feline leukemia virus (FeLV) contain a subgroup A virus (FeLV-A) that is essential for transmission. In contrast, FeLV-B is thought to arise *de novo* in the infected animal through RNA recombination events with endogenous FeLV transcripts, presumably through copackaging of RNA from endogenous FeLV and exogenous FeLV-A. Here, we report the complete genome sequences of two novel strains of FeLV-B (FeLV-2518 and FeLV-4314) that were isolated in the absence of FeLV-A. The *env* genes of these isolates have been characterized previously, and the 3′ recombination sites have been identified. We describe herein the 5′ recombination breakpoints of each virus. These breakpoints were found to be within the signal peptide of the *env* gene and the reverse transcriptase-coding region, respectively. This is the first report of a recombination site within the *pol* gene of an FeLV-B genome and the first genetic characterization of multiple independently arising FeLV-B isolates that have been identified without a functional FeLV-A ancestral virus.

## GENOME ANNOUNCEMENT

We recently reported the first known identification of feline leukemia virus subgroup B (FeLV-B) strains isolated without an accompanying subgroup A virus (1). Both isolates, FeLV-2518 and FeLV-4314, were fully infectious *in vitro*, questioning the previously held assumption that each FeLV-B isolate arises independently within an infected host and is not transmitted further to naïve hosts (2). As full-length, putatively functional, endogenous FeLV (enFeLV) proviruses have been described (3), and other enFeLV loci are known to be transcriptionally active (4–6), it is possible that the genomically intact enFeLV elements are transmitted in a horizontal manner between hosts. Such viruses would present as comprising FeLV-B only in subgroup analyses due to the shared receptor-binding properties of FeLV-B and enFeLV Env proteins (7, 8). The initial genetic characterization and receptor-usage assays of FeLV-2518 and FeLV-4314 indicated they were recombinant FeLV-B viruses. FeLV-2518 also copackaged a defective FeLV subgroup A (FeLV-A) transcript encoding a truncated FeLV-A Env protein (FeLV-2518[A]). The 3′ recombination site of FeLV-4314 was identified 200 bp downstream of the SU/TM cleavage site within the *env* gene, whereas that of FeLV-2518 was located 100 bp upstream of this motif (1). Both viruses therefore represent independent events, wherein a recombinant virus containing an unknown proportion of endogenously derived genomic sequence sequestered the originally present exogenous virus, potentially through the acquisition of exogenous long terminal repeats (LTRs) and the promoter elements within these domains (9). It was therefore of interest to sequence the remaining components of the FeLV-2518 and FeLV-4314 viral genomes. This would allow for the identification of the 5′ recombination sites and analyses of the relative proportions of each genome that are of endogenous and exogenous origins.

Viral RNA was isolated from cell-free supernatant from flat epithelial atypia cells and HEK293T cells infected with either FeLV-2518 or FeLV-4314. Multiple overlapping fragments of each viral genome were amplified by PCR using primers specific for both endogenous and exogenous FeLV. A sequence contig was then assembled and annotated by comparison with published enFeLV and exogenous FeLV viral genomes. The 5′ recombination breakpoint of FeLV-2518 was identified in the signal peptide-coding region of the *env* gene, whereas that of FeLV-4314 was located within the reverse transcriptase (RT)-coding region of the *pol* open reading frame. The genome of FeLV-4314 therefore contains a significant proportion of endogenously derived sequences, as the majority of Env and approximately half of the RT protein are encoded by enFeLV sequences. The endogenously derived component of a FeLV-B genome was thought previously to be limited to a central 250-bp region within the SU domain of *env* (10). Given the high nucleotide identity observed between enFeLV elements and exogenous genomes outside the *env* and LTR domains, FeLV-4314 may phenotypically be classified as a transmissible enFeLV isolate that has acquired exogenous LTR promoter elements. It is not known whether infection with this isolate would be detrimental to a host; endogenous retroviruses rarely display pathogenicities toward their wild-type host (11, 12), although the koala retrovirus, a virus that is in the process of endogenization, causes leukemia in koalas (13). Further studies into the transmission and pathogenic potential of this isolate will allow for the analysis of the respective contributions of the *env* gene and LTR regions in restricting the horizontal transmission of putatively functional enFeLV proviruses.

### Nucleotide sequence accession numbers. 

The FeLV-2518 and FeLV-4314 genomic sequences have been deposited in GenBank under accession numbers JF957361 and JF957363, respectively.
